# High resolution capillary western blot analysis of antibody reactivity in human visceral leishmaniasis

**DOI:** 10.1590/S1678-9946202567048

**Published:** 2025-07-18

**Authors:** Marcos Vinicius da Silva, Aldo Matos, Rafael Faria de Oliveira, Juliana Reis Machado e Silva, Malu Mateus Santos-Obata, Luciana de Almeida Silva-Teixeira, Dalmo Correa-Filho, Denise Bertulucci Rocha Rodrigues, Virmondes Rodrigues

**Affiliations:** 1 Universidade Federal do Triângulo Mineiro, Departamento de Imunologia, Microbiologia e Parasitologia, Uberaba, Minas Gerais, Brazil; 2 Universidade Federal do Triângulo Mineiro, Centro de Educação Profissional (Cefores), Uberaba, Minas Gerais, Brazil; 3 Universidade Federal do Triângulo Mineiro, Instituto de Biologia e Ciências Naturais, Departamento de Patologia, Genética e Evolução, Uberaba, Minas Gerais, Brazil; 4 Universidade Federal do Triângulo Mineiro, Instituto de Ciências da Saúde, Departamento de Medicina Interna, Uberaba, Minas Gerais, Brazil; 5 Universidade de Uberaba, Laboratório de Biopatologia e Biologia Molecular, Uberaba, Minas Gerais, Brazil

**Keywords:** Visceral leishmaniasis, Antibodies, Western blot

## Abstract

Visceral leishmaniasis (VL) is a potentially fatal disease, occurring in 76 countries, 12 of which are located in the Americas, with approximately 3,500 new cases annually registered in Brazil. Active visceral leishmaniasis is characterized by an intense inflammatory reaction with a low cell-mediated immune response and a high production of specific and non-specific antibodies. Antibodies are not associated with effective protective mechanisms but have been used widely in diagnostic tests. In this study, we analyzed the immunoglobulin G (IgG) response against crude antigens of *Leishmania infantum* by using automated western capillary blot in patients with active and clinically cured VL, individuals residing in an endemic area and patients with Chagas disease. The method allowed an accurate analysis of the antibody response. Our results demonstrated that antibody reactivity to *L. infantum* antigens in the 20–142 kDa range effectively distinguished active VL from clinically cured cases and Chagas disease, although these antigens were not exclusively recognized by patients with active VL. The automated western capillary blot proved to be a useful tool for differentiating patients with active VL from individuals living in endemic areas and those with Chagas disease, highlighting its potential application in serological diagnostics.

## INTRODUCTION

Visceral leishmaniasis (VL) is a potentially fatal disease, occurring in 76 countries, 12 of which are located in the Americas, with 96% of the cases registered in Brazil. The etiologic agent in the New World is a protozoan of the family *Trypanosomatidae*, *Leishmania infantum*, which multiplies in the cells of the mononuclear phagocytic system. Of those infected, about 90% are asymptomatic, but there is an estimated report of 20,000 to 40,000 deaths per year^1^. Clinical manifestations include fever, hepatosplenomegaly, pancytopenia, weakness, hemorrhagic manifestations, weight loss with progressive signs of malnutrition, tachycardia, diarrhea, and dry cough^2^. The most severe cases seem to be related to the profile of the immune response developed by the host and to the age of the patient, being more frequent in children in association with malnutrition.

The immune response in asymptomatic individuals is predominantly cellular, detected via a positive skin test^3^. Patients who develop classic symptoms have a deficiency in the Th1 response with reduced production of IL-2, IL-12, and IFN-γ, which recovers upon treatment^3,4^. In patients that suffered from a recent infection, the low production of IFN-y in response to IL-12 is associated with illness and seems to result from the modulating activity of IL-10^5,6^. The inflammatory response with the participation of TNF-α in synergism with IFN-γ has been pointed out at the same time as being capable of destroying the parasite and being responsible for tissue damage^3^. An inflammatory response involving IL-17 has been identified as protective against infections with intracellular pathogens, such as *Leishmania sp* ^7^. More recently, IL-6, an interleukin involved in directing the production of IL-17, has been associated with severe forms of VL^8^.

Active visceral leishmaniasis is characterized by an intense inflammatory reaction and suppression of the immune response by immunomodulatory mechanisms, such as those mediated by regulatory T cells (Tregs), IL-10, and TGF-β. High plasma levels of TGF-β and IL-10 are associated with a high parasite load in Indian patients, as well as their production by peripheral blood mononuclear cells (PBMCs) under stimulation by *Leishmania donovani* antigens^9-11^. However, no such association was observed between VL and the accumulation of Tregs, i.e., CD4+CD25+ T cells in the blood or spleen of Indian patients^10^. Furthermore, the antibody IgG1 was implicated in recurrence of human VL^12^ and susceptibility to canine kala-azar^13^.

The humoral immune response in VL has been studied predominantly as a means of diagnosis, due to its feasibility and lower cost. Methods include indirect immunofluorescence and enzyme immunoassays using crude or recombinant antigens, of which the most studied is rK39^14^. More recently, rapid test platforms with immunochromatography and biosensors have been introduced and are being developed^15^. Studies of the antibody response in VL indicate a substantial production of antibodies in individuals with the classic form of active VL and a low response in those already successfully treated, with asymptomatic infections, or presenting pathogen-related infections, such as Chagas disease^16^. The cross-reaction between VL and Chagas disease can be explained by the similarity of antigens between these parasites, which belong to the same family, *Trypanosomatidae*. The identification of specific antigens that can discriminate the clinical forms of VL and reduce cross-reactivity with other infections such as Chagas disease is important and urgent in endemic areas. At this point, western blot is a classic method to identify antibody repertory in VL^17^ *.* However, it presents great variability from between runs and is labor-intensive and time-consuming. Recently, a fully automated high-resolution capillary western blot has been developed, which incorporates critical validation steps to identify and minimize the sources of error and variability throughout^18^.

This study advances the analysis of humoral immune response evaluating the anti-*L. infantum* antigen IgG using the automated capillary-based western blot for the first time among patients with active and clinically cured VL, subjects residing in an endemic area, and patients with Chagas disease.

## MATERIALS AND METHODS

### Subjects

A total of sixty-seven serum samples were analyzed in this study, forty-five of which were from patients with clinical symptoms of VL whose diagnosis was confirmed by parasitological examination or serological testing combined with a specific therapeutic response. Healthy residents from endemic areas without a history of VL or Chagas disease, aged from one to 67 years, residents of the Paracatu municipality, Northwest region of the Minas Gerais State, Brazil, were analyzed. All patients were asked to come for a follow-up conducted by the assistant physician and the epidemiological surveillance service, which included a clinical evaluation and a serological examination using the indirect immunofluorescence test. Of these, 15 patients had acute VL (six were males). Additionally, thirty patients were classified as clinically cured from VL. These individuals had completed treatment for VL and no longer exhibited clinical symptoms of the disease, although they may still have detectable antibodies. Their clinical cure was confirmed by follow-up evaluations, including clinical assessments and serological tests. Among the 30 clinically cured VL individuals (17 males), 10 were treated less than two years ago (five males), 11 were treated between two and four years ago (six males), and nine were treated more than four years ago (six males). Seventeen serum samples from healthy donors (seven males) and five from patients with Chagas disease (three males) served as control groups. The group compositions are presented in Table 1. All subjects were nonreactive for HIV antibodies. The sixty-seven samples were randomly selected from a study that recruited one hundred ninety-one patients form the Paracatu municipality. The sample calculation was established based on 172 existing serum samples, with a confidence level of 95% and a 10% margin of error. This study was approved by the UFTM Research Ethics Committee under the N° 58301516.8.0000.5154.

**Table 1:** Distribution of study participants by group, sample size, and sex.

Group			n	Male/Female
Visceral leishmaniasis			45	23/22
	Acute VL		15	6/9
	Clinically cured VL		30	17/13
		Clinically cured VL (<2 years)*	10	5/5
		Clinically cured VL (2–4 years)*	11	6/5
		Clinically cured VL (>4 years)*	9	6/3
Chagas disease			5	3/2
Healthy donor			17	7/10

* All patients belong to the group with visceral leishmaniasis and the subgroup clinically cured VL.

### Obtaining and processing samples

After the patients signed the Free and Informed Consent Form, blood samples were collected from them via a Vacutainer^®^ system with separator gel and processed to obtain serum, which was separated and frozen at −20 °C for antibody analysis.

### Parasite culture and antigen preparation

The *L. infantum* strain MCER/BR/79/M6445 was cultivated at 28 °C in Schneider's medium (Sigma) supplemented with 20% inactivated fetal bovine serum, 1% sodium pyruvate, 1% L-glutamine, 750 mg/L calcium carbonate and 40 μg/mL gentamicin. The parasites were cultured at 28 °C in plastic bottles for cell culture (Corning, Lowell, MA).

Upon reaching the stationary phase, the parasites were collected and centrifuged at 800 x*g* for 30 min at 4 °C. The parasites were washed three times with an incomplete RPMI medium. For antibody detection, the parasites obtained by centrifuging the culture were resuspended in Tris-HCl buffer, pH 7.2, containing a protease inhibitor cocktail (COMPLET Roche, Niederlenz, Switzerland) and vortexed vigorously for five minutes. The mixture was centrifuged at 4000 x*g* for 30 min at 4 °C and the supernatant was filtered through a 0.22 μm membrane (Millipore, Molsheim, France). Protein concentration was determined with a spectrophotometer (Nandrop – Thermo Fisher, Whatman, MA, USA) and estimated at 2.8 mg/ml. The antigen was aliquoted and stored at −70 °C.

### Automatized capillary western blot

The reactivity of antibodies from patients and controls to *Leishmania* antigens was determined using the Wes Protein Sample automatic capillary western blot system (San Jose, CA, USA). *L. infantum* antigens at a concentration of 500 μg/mL were briefly combined with 1 part 5× Fluorescent Master Mix (containing 5× sample buffer, 5× fluorescent standard, and 200 mM DTT) and heated at 95 °C for 5 min. After this denaturation step, the prepared samples, blocking reagent, primary antibodies (patient serum diluted 1:250 in PBS pH 7.2), HRP-conjugated secondary antibodies (anti-human IgG diluted 1:250 in PBS pH 7.2), and chemiluminescent substrate were dispensed into designated wells in an assay plate. A biotinylated ladder (12 kDa to 230 kDa) provided molecular weight standards for each assay. After plate loading, the separation electrophoresis and immunodetection steps took place in the fully automated capillary system in a 2-hour run. The anti-Leishmania antibodies from the serum samples bound to Leishmania-derived proteins were proportional to the signal produced from the HRP-labeled secondary antibody, which is digitally recorded upon a chemiluminescent reaction. The Compass Simple Western software (version 5.0.1, ProteinSimple) was used to automatically calculate height (chemiluminescence intensity), area, and signal/noise ratio, as well as to capture the digital image of the capillary chemiluminescence.

### Data analysis

Data were analyzed using the GraphPad Prism (version 8.0, GraphPad Software, La Jolla, CA, USA) and the StatView software (version 4.0, Abacus, USA). Normality was checked using the Kolmogorov–Smirnov test. The Student *t-*test or the Mann-Whitney U test were used to compare two groups at the same time point, and ANOVA (Analysis of Variance) or the Kruskal-Wallis test were used to compare three or more groups. The results were expressed as mean ± standard deviation with a significance level of 0.05.

## RESULTS

### Active VL patients present higher antibody reactivity to *L. infantum* antigens than clinically cured individuals

**Figure 1:**
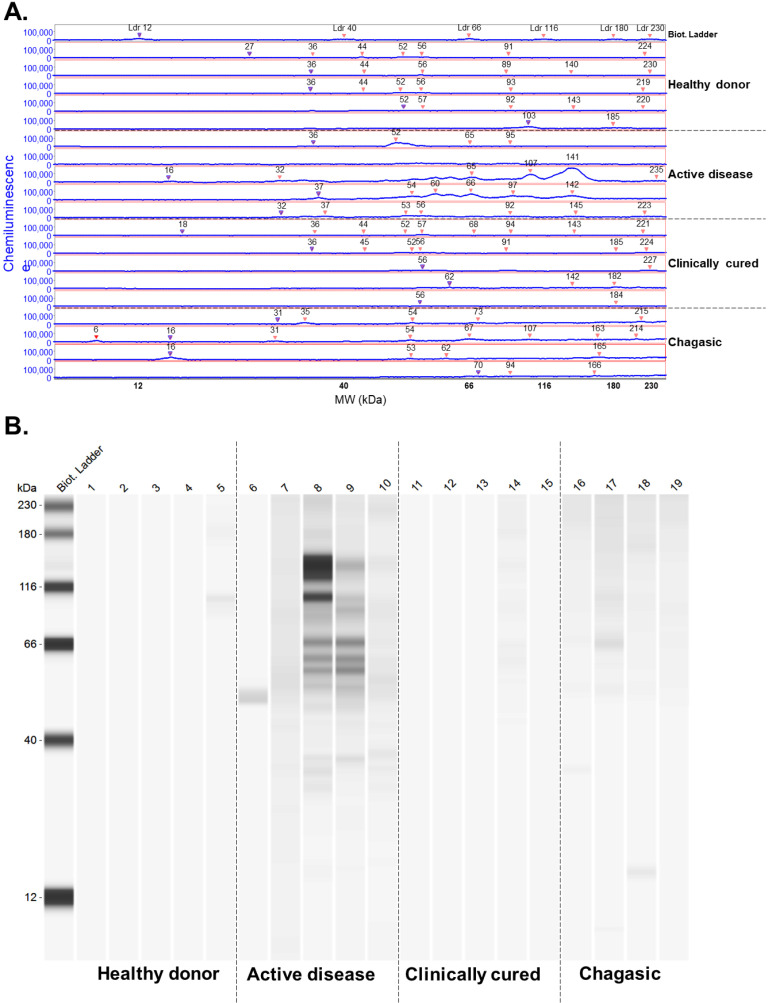
IgG antibody reactivity to *L. infantum* antigens from healthy donors, patients with active VL, clinically cured VL, and Chagas disease: (A) intensity of the IgG antibody reactivity to *L. infantum* antigen in the automated western blot in healthy individuals, patients with active infection, cured infection, and Chagas disease; (B) representative reactive bands.

**Figure 2:**
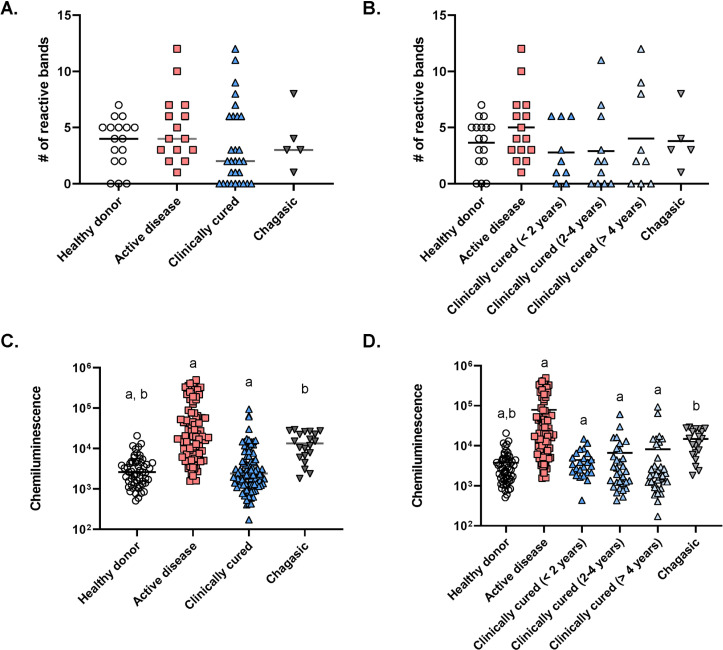
Acute VL patients present more intense IgG antibody reactivity to *L. infantum* antigens than clinically cured VL: (A) and (B); number of IgG-reactive WB bands; (C) and (D): number of IgG-reactive WB bands.

The serum samples analyzed exhibited IgG antibody reactivity against many *L. infantum* antigen fractions with molecular masses ranging from 6.3 to 234 kDa (Figure 1). The number of reactive western blot bands per serum sample was similar among healthy donors; those with acute VL; with clinically cured VL, regardless of time since they were cured; and patients with Chagas disease (Figures 2A and 2B). It is interesting to note that bands with a molecular weight of 142 were reactive only to serum from patients with VL (Figure 1A). Furthermore, the global intensity of antibody reactivity to *L. infantum* antigens was significantly higher in active VL than clinically cured VL, regardless of time since clinical cure, and healthy donors were similar to patients with Chagas disease (p < 0.0001, Kruskal-Wallis test, Figure 2C). Moreover, the mean intensity of the global IgG reactivity to *L. infantum* antigens is reduced in VL patients clinically cured for less than two years compared to 2–4 and > 4 years after cure, but those present a bimodal pattern despite the majority of IgG reactivity reduction (p < 0.0001, Kruskal-Wallis test, Figure 2D).

The IgG antibody reactivity to *L. infantum* antigens detected by capillary western blot using a chemiluminescent (CL) reaction. CL intensity (arbitrary unit) was proportional to antigen-antibody interaction. *p < 0.05: Kruskal-Wallis test followed by Dunn's post hoc test. Horizontal lines represent the median, bars represent the 25^th^-75^th^ percentiles and vertical lines represent the 10^th^–90^th^ percentiles.

### 20 kDa to 120 kDa and > 200 kDa *L. infantum* antigens are better to discriminate acute VL from healthy donors and clinically cured patients

**Figure 3:**
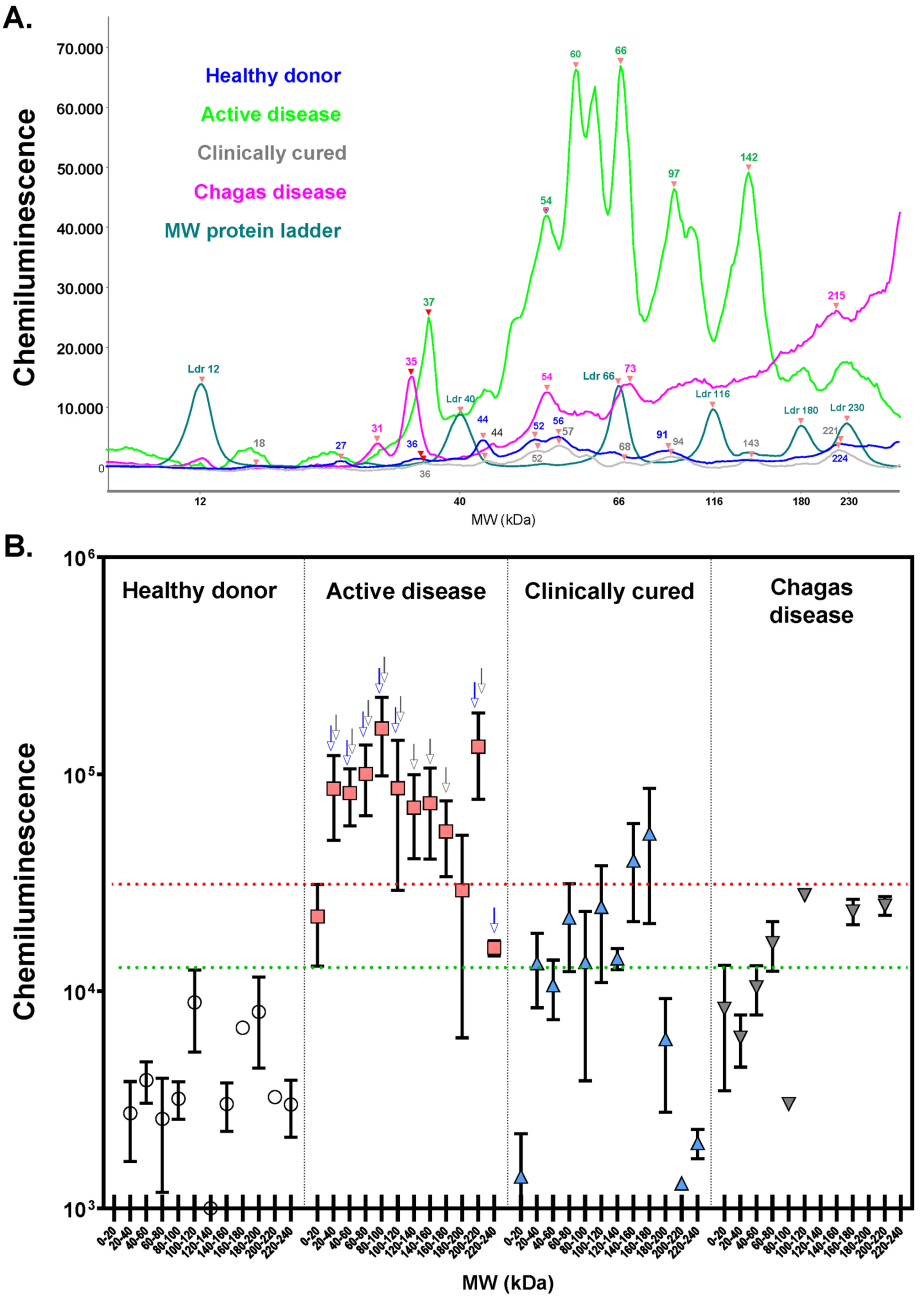
IgG reactivity to 20-120 kDa *L. infantum* antigens presents better results in discriminating active VL: (A) representative electropherogram of serum IgG-reactivity to *L. infantum* antigens. Numbers point to MW in kDa of each recognized antigen; (B) intensity of IgG reactivity to *L. infantum* antigens for each 20kDa interval of MW. The IgG antibody reactivity to *L. infantum* antigens detected by capillary western blot using a chemiluminescent (CL) reaction. Chemiluminescence intensity is proportional to antigen-antibody interaction. Blue arrows point to the MW interval with significantly different intensities compared to clinically cured patients. Gray arrows point to the MW interval with significant different intensity compared to patients with Chagas disease. *p < 0.05: Kruskal-Wallis test followed by Dunn's post hoc test. Horizontal lines represent the median, bars represent the 25^th^-75^th^ percentiles and vertical lines represent the 10^th^-90^th^ percentiles.

The analysis of the *L. infantum* antigens was grouped in 20 kDa intervals (less than 20 kDa to 220–240 kDa). A representative plot of antigens recognized by IgG for a patient of each group and MW (molecular weight) protein ladder is shown in Figure 3A. The intensity of IgG antibody reactivity against *L. infantum* antigens was significantly higher in patients with active VL than in healthy donors for all intervals analyzed. Serum IgG from active VL patients recognized antigens with 20–180 and 200–240 kDa with higher intensity. Of these, antigens with MW ranging from 20 to 120 kDa are recognized with significantly greater intensity by sera from patients with acute VL than by sera from patients with clinically cured VL (p < 0.05, Kruskal-Wallis test, Figure 3B, blue arrows) and individuals with Chagas disease (p < 0.05, Kruskal-Wallis test, Figure 3B, gray arrows). Furthermore, serum from active VL patients recognized 120–180 kDa antigens more strongly than serum from patients with Chagas disease (p < 0.05, Kruskal-Wallis test, Figure 3B, gray arrows).

High MW *L. infantum* antigens (>200 kDa) are more intensely recognized in serum from active VL patients than after clinical cure, although antigens >220kDa are also recognized with high intensity in serum from patients with Chagas disease, albeit at a lower intensity. This cross-reactivity in Chagas disease patients should be considered in future studies comparing seroreactivity between active VL and Chagas disease, especially when using these proteins, due to the potential for cross-reactivity (p < 0.05, Kruskal-Wallis test, Figure 3B, gray arrows).

### Clinical cure of VL is accompanied by modifications in the *L. infantum* antigen recognition pattern

**Figure 4:**
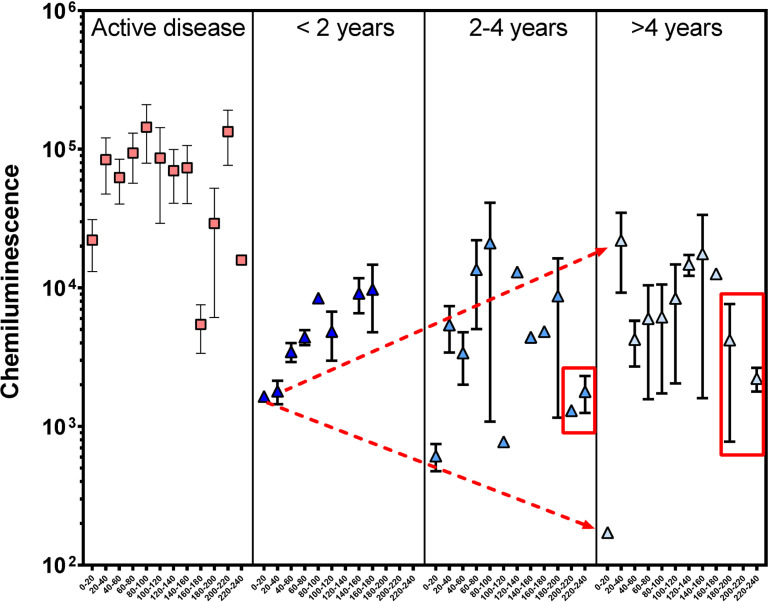
IgG reactivity to <20, 20–40 and >200 kDa *L. infantum* antigen after clinical cure are modified through time. The intensity of IgG reactivity to *L. infantum* antigens for each 20kDa interval of MW. The IgG antibody reactivity to *L. infantum* antigens detected by capillary western blot using a chemiluminescent reaction, whose intensity is proportional to antigen-antibody interaction. The dotted red arrows indicate the variation of IgG recognition intensity to > 20 and 20–40 kDa *L. infantum* antigens. The red box indicates IgG reactivity after two years of clinical cure of LV. Horizontal lines represent the median, bars represent the 25^th^–75^th^ percentiles and vertical lines represent the 10^th^–90^th^ percentiles.

The clinical cure of VL is accompanied by a significant reduction of the intensity of IgG antibody reactivity against *L. infantum* antigens in all MW intervals analyzed. However, the IgG reactivity to very low MW antigens (<20kDa) reduces with time after cure, while reactivity to 20–40kDa is increasingly higher (p < 0.05, Kruskal-Wallis test, Figure 4, dotted red lines). Moreover, reactivity to antigens with more than 200 kDa is only detectable after two years of clinical cure, albeit at a lower intensity than in active VL patients (Figure 4, red boxes).

## DISCUSSION

Visceral leishmaniasis (VL) continues to present significant challenges in both diagnosis and treatment. Our study highlights the critical differences in antibody reactivity to *Leishmania infantum* antigens in active VL patients compared to those who are clinically cured or suffer from Chagas disease. The findings show that, while active VL patients exhibit higher antibody reactivity, clinical cure is associated with a reduction in antibody diversity, with an increase in reactivity to higher MW antigens. This reduction in antibody reactivity over time after treatment suggests that immunological memory and the presence of persistent but non-pathogenic antibodies play key roles in the resolution of the disease.

One of the most challenging problems in the diagnosis of VL is the correct identification of mild symptomatic or asymptomatic cases since the current methodologies are flawed^19^. Furthermore, one of the most common problems in real-life diagnosis is the merged endemic areas for leishmaniasis and Chagas disease and the shared immunoreactivity to several *Trypanosoma cruzi* and *Leishmania* spp. antigens^20–22^.

The classical studies evaluating the patterns of antibody repertoire in VL are mostly based on immunoblotting. Despite the valuable contributions to basic research and diagnosis^23^, western blot is a laborious technique, which makes it difficult to compare studies conducted in different locations or even in various serial evaluations. One of the most recent evolutions of immunoblot is an automated capillary-based western blot technology^18^. This technique greatly reduces the variation caused by the multi-step manual approach by employing fully automated steps in a closed system. Furthermore, the chemiluminescent detection of antigen-antibody interaction enables a reliable and comparative quantitative analysis with high throughput. This technology has been used to detect anti-drug antibody responses^24,25^, in vaccine development^26,27^, evaluation of protein purification^28^ and quantification^29^, and antibody reactivity pertaining infectious diseases such as COVID-19^30^ but not parasitic diseases.

One of the reasons that can explain the difficulties in VL diagnosis is the polyclonal activation of B cells with hypergammaglobulinemia and the production of parasite-specific and nonspecific antibodies^31,32^. Additionally, VL patients present different patterns of Fc N-glycosylation associated with disease severity^33^. The first studies evaluating antibody reactivity to *L. infantum* antigens revealed a broad spectrum of antibody-targets, especially 12–120kDa antigens^34,35^. The analyses of VL sera reactivity to *L. infantum* showed antibodies to most of the antigens present in the parasite extract^34,36^. Our study demonstrates that VL patients produce antibodies against several *L. infantum* antigens. Patients with active VL produce higher amounts of antibodies directed to several *L. infantum* antigens and the clinical cure is associated with a reduction of antibody diversity and the rise of antibodies directed to high MW antigens. However, the number of *L. infantum* antigens recognized by the Chagas disease serum is high and could explain the cross-reaction observed in several serological tests employing total or purified antigens^22,37^.

Our study found that all active VL patients evaluated presented reactivity to *L. infantum* antigens. While very low MW antigens (<20 kDa) appear useful for distinguishing non-infected individuals, the reactivity to these antigens is also observed in clinically cured VL patients and those with Chagas disease. Previous reports showed low MW antigens as potential targets for VL diagnosis^35,38^. However, the follow-up to ELISA (Enzyme-Linked Immunosorbent Assay) diagnosis indicates controversial results, with inaccuracy in detecting asymptomatic infection^39^. In contrast to previous studies, we demonstrated that clinically-cured individuals and asymptomatic patients present antibodies of >20 kDa antigens, which reduce with time. Based on these results, we can hypothesize that the evaluation of patients—regardless of the time after cure and using a low-sensitivity and non-quantitative technique—could be an important confusing factor in identifying potential *L. infantum* antigens for the diagnosis and follow up of patients.

## CONCLUSION

*L. infantum* antigens of 20–142 kDa showed the best performance in differentiating active disease from no disease, clinical cure, and Chagas disease, although not exclusively recognized by active VL patients. Even though this potential for discrimination was evidenced predominantly by the high intensity of the reaction, it is important to highlight that patient with Chagas disease did not react to these antigens. Furthermore, the low cross-reactivity observed may be overcome by using more highly diluted serum in future detection tests. It is worth highlighting that the dilutions used in this study aimed to demonstrate the broadest spectrum of the antibody repertoire and potential cross-reactions. The findings presented here must be interpreted considering the number of patients included in this study. Other studies point to this MW range of *Leishmania* antigens as being promising for diagnosis in humans and dogs^17,35,36,40^. Moreover, we demonstrated that: 1) cross-reactivity is an important factor to consider; 2) our quantitative approach indicates that antigens could be used in optimized diagnostic tools focused on reducing the impact of cross-reactivity, such as modified biosensors^20^. These findings emphasize the value of using quantitative and automated techniques in future VL diagnostics, potentially leading to the development of tools that can differentiate between active disease, clinical cure, and cross-reactivity from other diseases like Chagas.
